# Facts vs. Figures

**Published:** 1850-05

**Authors:** 


THE MEDICAL EXAMINER.

PHILADELPHIA, MAY, 1850.

### FACTS *VS.* FIGURES.

Figures, Mr. Editor, are not statistics, nor yet facts, although it is a very common notion that they are both. They are too often little better than figures of speech, and like many other such forms of expression, impose themselves for truths upon the minds of the unwary. When they stand for definite and ascertained realities, they form the best possible medium of comparison between different classes of phenomena or ideas; but when they are only the translation of an hypothesis, nothing can be more mischievous and deceptive. They become the lion's skin to the ass concealed within them, and give a semblance of dignity and strength to things the weakest and most vulgar.

I was led into these reflections by the curious calculations contained in two portions of your last number, where it is demonstrated arithmetically, by one writer, that we create annually less Doctors, by from 20 to 35 per cent., than are demanded, and by another, that the supply is at least 50 per cent. too small. Here, to be sure, is a serious discrepancy in the mathematics of the two authorities, but it is nothing to an omission of which both are guilty. Perhaps a reluctance to reveal the whole startling truth may have caused a suppression of this essential element in the calculation. It was sad enough to think that with all the efforts of the schools from Maine to California to hurry out half-made, underdone physicians, there was still a lack of from one-third to one-half of the required number of these recruits. What would have been the despair of the grinders in these factories had they been told how much further still they were from the possibility of satisfying the demand for their commodity. Their patriotic souls would have sunk within them at the thought. But even at the risk of renewing their grief, we must disclose what your too scrupulous statisticians charitably concealed, for we cannot suppose that it escaped their sagacity.

If, in this year of grace 1850, twenty-five hundred graduates are required, and only fifteen hundred are created, the manufacture of doctors is short by one thousand. But the 2500 graduates are required to supply the demand of *this year alone. Whence is the deficit of preceding years to be made up?* If we create a thousand too few doctors during this year, we made at least nine hundred too few last year, and so on for previous years; the aggregate of these deficiencies being added to that of the present year, would give a total of three to five thousand required in 1851 for equalizing the supply and demand!

A moment's reflection will satisfy you, Mr. Editor, that this conclusion, absurd as it certainly is, is the legitimate consequence of the argument stated in your last number; by itself it demonstrates the foundation of that argument to be unsound. So that the schools have already good reason to take courage, and rejoice that their unselfish labors are not quite so inadequate to the object they seem to have in view, as the articles you published do clearly imply.

But, after all, what ground is there for the assumption out of which the whole calculation proceeds? It is simply that in "the city and suburbs of Philadelphia," the number of medical men is 497, and the population within the same limits 350,000. We are not informed on what authority the estimate is given, and must consequently regard it as nothing more than a supposition, which may be true or false, but which certainly offers no proof of a claim to the former character. Yet it is assumed by the one writer, and adopted by the other, as if it were an ascertained fact.

The next step in the argument is another assumption: the one writer considering that there are 31,250 doctors in the United States, and the other 26,875, the former supposing the hypothetical ratio of Philadelphia to extend to the whole country, and the latter the still more gratuitous ratio of one physician to every 800 souls. The first named gentleman, indeed, attempts to sustain his deduction by stating that a publishing house in this city has the addresses of more than 30,000 physicians; but every one must perceive that such a list can by no possibility be accurate, however well it may answer for business purposes.

In all of these estimates we see no account taken of the emigration of physicians to this country from Europe. One writer, to be sure, alludes to the point, and passes it by with the remark that we gain by immigration about as many as we lose by emigration!

Why, the graduates or licentiates of British and German colleges may be counted in this very city by scores, and New York is pretty well furnished besides with adventurous Frenchmen driven from their native country by the overcrowding of their profession. When it is known abroad that we need annually a thousand more doctors than we can make, a very avalanche of greedy and needy medicos will roll upon our quays. Then, if ever, may we take up Abernethy's unconscious soliloquy, and exclaim, "Heaven help ye, what's to become of ye all!"

The per centage of deaths, withdrawals fiom the profession, &c., which, according to the articles in question, create an annual deficit and demand more than equal to what is required by the increase of the population, calculated as it is on the presumed number of physicians, can have of course no greater validity than this latter, which in its turn rests on the assumed population, and the assumed number of physicians in Philadelphia and its suburbs. The old story, Mr. Editor, of the earth resting on the elephant, the elephant on the tortoise, and the tortoise upon—what?

Now, I wish distinctly to remark, that it is not my purpose to deny the numerical statements given above, but only to show that there is no evidence of their foundation in fact, and to contend that without such a foundation it is unfair to employ them as arguments in discussing a question which interests the dignity and prosperity of the medical profession. For if there is no such disparity as that alleged between the demand and supply of physicians, but, on the contrary, an actual excess of the latter, the profession as a body must suffer in every respect by the crowding of its ranks.

There are no figures at present by which this question can be settled, unless we compare our own with foreign countries. The only domestic data we have, are the population and the annual number of graduates. Now, unless it can be shown that there is something to account for the difference, why should the proportionate number of our graduates exceed that of other civilized counties—of France, for example? If her population of thirty-five millions, her colonies, her army of half a million, and her extensive marine, call for not so many as five hundred physicians annually, why should our population of twenty-two millions require fifteen hundred physicians to be made every year, a proportion nearly five times as great as the other? If these data and conclusions are incorrect, I shall be heartily glad to have their errors pointed out, but until then, I shall be constrained to believe that the striking disparity here indicated is of itself a sufficient proof that we already graduate too many physicians.

But the truth is, Mr. Editor, that we have no need of statistics of one sort or another to solve this problem perfectly; were it submitted to a jury of practitioners having no interest in the multiplication of doctors, they would decide it without leaving the box. Every man of them would feel that his own experience was conclusive in the matter, and that his hard struggles to earn a livelihood showed beyond a shadow of doubt, that there are many more practitioners of medicine than are required. The sentiment is universal in our large cities, and from the country we hear no complaints of the paucity of physicians. These facts defy controversy, and are worth more than all the adverse statistics in creation.

We shall be told, however, that the pressure is not from physicians, but from quacks, who usurp the place which educated men should occupy. Very well, extirpate the quacks, and then let us see how the account stands; but, until that is done, permit me to say that it makes very little difference whether I starve through a knave's impudence, or more genteelly die of inanition through the successful rivalry of a brother M. D. It really seems, Mr. Editor, as if one could hardly be in earnest, in using such language as one of the writers whom you quote. To think of sending forth some ten scores of graduates with the cheerful information that they are to make their bread by "superceding," not open quacks alone, but ignorant doctors into the bargain! And how? Are these owls to be driven to their dens by the light of science? Rather will they extinguish that light by absorbing all the oil which was counted upon for keeping it alive. How is it in our largest and most enlightened cities? Why, the very flower of the profession in science, learning, manners, social position, in everything that commands respect—these are the very men who daily receive their *congé*, and at the bedside where wisdom and conscience and benevolence sat enshrined in their persons, vulgarity, ignorance and knavery play their antics, and are caressed by their enlightened patrons. I should be glad to have the recipe for "superceding" such men. But it is not forthcoming. It of all things certainly is not—*Take:*

"*Three thousand graduates,*" *&c.*, as the author hints. The working of that prescription would be just the reverse of what is desirable. It would make quacks; it would multiply them, rather, for the one in operation now is making them, and that rapidly, as every one who will open his eyes must see. Men without education made students of medicine, and half educated then, learn to look upon medicine as a trade, and if they cannot drive it profitably in the regular way, and they cannot, they will become knavish as a matter of course.

It is of no avail to calculate how many physicians there ought to be for the whole population, until you ascertain how many of the latter prefer quackery to medicine ; for in estimating the relation of supply and demand, every quack represents a physician. But our statistical friends do not seem to have thought of this. I commend it to them for the rectification of their arithmetic.

I remain, &c. &c. Y. Z.

